# DNA electroporation of HIV Env elicits robust T cell responses and memory B cell responses with muted serum antibody levels that can be boosted with recombinant protein

**DOI:** 10.1016/j.vaccine.2026.128487

**Published:** 2026-03-21

**Authors:** Stephen C. De Rosa, Ronnie M. Gravett, William O. Hahn, Manuel Villaran, Yunda Huang, Lorel Schmitzberger, David Montefiori, Elizabeth Domin, Georgia D. Tomaras, Jack Heptinstall, Kelly E. Seaton, Guido Ferrari, Gabriel Ozorowski, Andrew B. Ward, Wen-Hsin Lee, Lara van der Maas, Laura Polakowski, Ian Frank, Hong-Van Tieu, Spyros A. Kalams, Nina Marie G. Garcia, Jinwei Huang, Sukanya Ghosh, Mansi Purwar, Dan Kulp, Laurent Humeau, Peter D. Kwong, M. Juliana McElrath, Lawrence Corey, David Weiner

**Affiliations:** a Vaccine and Infectious Disease Division, Fred Hutchinson Cancer Center, Seattle, WA, USA; b Laboratory of Medicine and Pathology, University of Washington, Seattle, WA, USA; c Division of Infectious Diseases, Department of Medicine, Heersink School of Medicine, University of Alabama at Birmingham, Birmingham, AL, USA; d Department of Medicine, Division of Allergy and Infectious Disease, University of Washington, Seattle, WA, USA; e Department of Global Health, University of Washington, Seattle, WA, USA; f Duke University Medical Center, Durham, NC, USA; g Department of Surgery, Duke University Medical Center, Durham, NC, USA; h Department of Integrative Structural and Computational Biology, The Scripps Research Institute, La Jolla, CA, USA; i National Institute of Allergy and Infectious Diseases, National Institutes of Health, Rockville, MD, USA; j Division of Infectious Diseases, Perelman School of Medicine, University of Pennsylvania, Philadelphia, PA, USA; k Laboratory of Infectious Disease Prevention, Lindsley F. Kimball Research Institute, New York Blood Center, New York, NY, USA; l Division of Infectious Diseases, Department of Medicine, Columbia University Irving Medical Center, New York, NY, USA; m Division of Infectious Diseases, Department of Medicine, Vanderbilt University Medical Center, Nashville, TN, USA; n Department of Pathology, Microbiology, Immunology, Vanderbilt University, Nashville, TN, USA; o Vaccine and Immunotherapy Center, The Wistar Institute, Philadelphia, PA, USA; p Inovio Pharmaceuticals, Plymouth Meeting, PA, USA; q Vaccine Research Center, National Institute of Allergy and Infectious Disease, National Institutes of Health, Bethesda, MD, USA; r Aaron Diamond AIDS Research Center and Departments of Medicine and Biochemistry and Molecular Biophysics, Vagelos College of Physicians and Surgeons, Columbia University, New York, NY, USA

**Keywords:** HIV-1 vaccine, DNA vaccine, Clinical trial, Neutralizing antibodies, B cell response, CD8+ T cell response

## Abstract

Broadly neutralizing antibodies against the HIV envelope protein exhibit the potential to prevent HIV-1 acquisition, a concept demonstrated both in non-human primate (NHP) challenge models and in human clinical trials. The use of DNA for vaccination, in combination with IL-12 and delivered via intradermal (ID)-adaptive electroporation (EP), has resulted in excellent cellular and humoral immunogenicity. HVTN 304 (NCT05828095) is a first-in-human, phase 1 clinical trial that evaluated the safety and immunogenicity of a novel vaccination regimen of a synthetic DNA-encoded stabilized HIV-1 Env native-like trimer (sD-NLT-AB05) adjuvanted with IL-12 DNA, either alone or in combination with a recombinant protein HIV-1 Env (Trimer 4571) adjuvanted with 3M-052-AF/Alum as a boost, in 20 participants without HIV. The DNA vectors were delivered via ID EP. The regimen did not induce autologous neutralizing antibodies in serum. Binding antibodies to Env were induced in over half of the participants receiving DNA only and in all participants who received the Trimer 4571 boost; the magnitude was increased by the second protein boost. The DNA-induced antibodies were primarily directed to the Env base; some Trimer protein-induced antibodies were directed to other regions of Env and the base. Env-specific CD4+ T cells expressing IFN-γ and/or IL-2 were detected in all participants, with CD8+ T cells detected in up to 75% of participants. The CD4+ T cell response included cells expressing IL-21. This study demonstrates that the DNA modality, in combination with a closely related heterologous boost, may be another modality to enable iterative testing of new vaccine regimens for HIV-1, as it primes a B cell response and a CD8+ T cell response without inducing high titers of serum antibody.

## 1. Introduction

In 2023, over one million people acquired human immunodeficiency virus (HIV), raising the global total to nearly 40 million living with HIV [1]. While biomedical prevention strategies such as pre- and post-exposure prophylaxis (PrEP/PEP) effectively prevent HIV acquisition, barriers such as low awareness, limited access, programmatic shutdowns, and inadequate adherence challenge their real-world effectiveness [2]. Durable protection through active immunization can overcome many of these barriers, underscoring the critical need for an effective HIV vaccine.

DNA vaccination has been a technology used over the last two decades to deliver candidate vaccines. Several such vaccines are in advanced clinical trials for infectious diseases and cancer [3–8]. The synthetic DNA modality has emerged as a promising approach for HIV vaccination. When combined with IL-12 DNA and delivered via intradermal electroporation (ID EP) along with protein boosts, it presents several advantages over traditional subunit protein vaccines, including the potential to elicit more robust cellular immune responses compared to protein vaccines alone, dose sparing of the protein vaccine, and the unique induction of cellular responses that synergize with humoral responses [4]. Manufacturing and development timelines are shorter with DNA compared to other vaccine modalities based upon recombinant proteins or viral vectors, and DNA vaccines are generally more thermostable than mRNA vaccines [9]. Prior clinical studies have demonstrated the safety of DNA-encoded HIV Env immunogens based upon gp120 monomers delivered via EP, with consistent induction of antibody and T cell responses [4,10]. Native-like trimer (NLT) Env proteins are key targets for HIV vaccine development [11,12]. Preclinical studies have shown that recombinant NLTs, which mimic the ‘native’ Env protein on the HIV virion surface by maintaining the trimeric structure of Env, display epitopes recognized by all broadly neutralizing antibody (bnAb) lineages capable of neutralizing HIV. Similarly, Env trimers conceal many non-neutralizing epitopes that do not directly mediate protection from HIV acquisition [12].

The sD-NLT-AB05 encodes an Env trimer derived from the HIV-1 clade A strain BG505, the canonical HIV Env trimer extensively evaluated in preclinical studies and tested as a vaccine immunogen delivered via recombinant protein [13]. The original BG505 gp140 format has been modified through directed mutations to produce a more stable NLT that exposes epitopes targeted by neutralizing antibodies (nAbs) while occluding epitopes targeted by non-neutralizing antibodies (“distracting” epitopes) [14,15].

Another BG505 derivative with different stabilizing mutations, Trimer 4571, has been evaluated in clinical trials as recombinant protein adjuvanted with alum [16]. The 3M-052-AF adjuvant, a toll-like receptor (TLR) 7/8 agonist, has been shown to generate strong antibody responses in humans vaccinated with BG505 SOSIP.664 gp140 [13]. The combination of an Env trimer with 3M-052-AF is generally safe and well tolerated [13]. When adjuvanted with alum, Trimer 4571 did not elicit detectable levels of autologous neutralizing antibodies in serum [16]. However, memory B cells encoding antibodies capable of neutralization were evident in vaccinated participants [17]. In contrast, the closely related BG505 trimer elicited detectable nAbs in serum, and when administered with 3M-052-AF/Alum, antibodies capable of potent strain-specific neutralization could be isolated from memory B cells, suggesting that adjuvants may play a key role in the levels of serum antibodies [13].

The combination of a DNA prime followed by a protein boost drives a robust response to gp120 monomeric Env, compared to protein alone [18]. As the immune response to monomeric gp120 is distinct compared with the immune response to intact HIV Env trimers, whether this same concept applies to trimeric HIV Env is unknown.

This first-in-human, phase 1 clinical trial evaluated the safety and immunogenicity of a novel vaccination regimen utilizing a synthetic DNA-encoded stabilized HIV-1 Env NLT (sD-NLT-AB05) [19] adjuvanted with IL-12 DNA administered via ID EP, either alone or in combination with HIV-1 Env Trimer 4571 adjuvanted with 3M-052-AF/Alum as a boost delivered intramuscularly (IM).

## 2. Material and methods

### 2.1. Study design

HVTN 304 (NCT05828095) is a randomized open-label Phase 1 trial to examine the safety and immunogenicity of INO-6160 (synthetic DNAs encoding a native-like HIV Env Trimer and Interleukin-12) delivered via ID EP, alone (Group 1) or in a prime-boost regimen with VRC HIV Env Trimer 4571 adjuvanted with 3M-052-AF/Alum (Group 2) delivered IM, administered as separate doses to healthy participants without HIV. See Table 1 for Study Schema.

Twenty participants were enrolled across 3 clinical research sites in the United States: Nashville, New York, and Philadelphia. Participants were eligible if they were between 18 and 55 years of age, living without HIV-1, in good overall health, and considered not likely to acquire HIV during the study period.

### 2.2. Ethics

A single institutional review board (IRB) (Advarra) approved the study protocol, with review by the local IRB as indicated. All participants provided written informed consent. The HVTN Safety Monitoring Board provided safety oversight.

### 2.3. Safety assessments

Safety was closely monitored throughout the study. Solicited adverse events (AEs) typically observed following vaccination were observed and documented for each participant for the 14 days following each vaccination. Unsolicited AEs were observed and documented over a period of 30 days after each vaccination and captured in the clinical database. A limited set of AEs, including serious adverse events (SAEs), adverse events of special interest (AESIs), medically attended adverse events (MAAEs), and AEs leading to early participant withdrawal or early discontinuation of study vaccine administration, were observed and documented for 12 months following the last vaccine administration.

### 2.4. Laboratory methods

Assays were conducted on specimens obtained at months 1.5, 3.5, and 6.5 corresponding to 2 weeks after the 2nd, 3rd, and 4th vaccinations, respectively.

#### 2.4.1. Binding antibody multiplex assay (BAMA)

Serum responses to AB05 and Trimer 4571 were measured by coupling directionally biotinylated His-Avi-tagged HIV Env trimers to NeutrAvidin-coated fluorescent beads [13,20–22]. To detect base-specific responses, GNL-coated beads were used to capture untagged BG505 MD39.3 trimers (wild-type and BaseKO), thus orienting the trimer base outward via gp120 glycan binding. Serum samples (starting dilution 1:50) were incubated in duplicate with the multiplexed beads in 96-well plates. Mean fluorescence intensities (MFI) were recorded with a BioRad 200 system, with background subtraction using blank wells. Trimer 4571 was provided by the Vaccine Research Center (VRC) at the US National Institute of Allergy and Infectious Diseases (NIAID) and AB05 was provided by the Wistar Institute.

#### 2.4.2. Neutralizing antibodies assay

Neutralization titers (ID50 and ID80) were determined by the reduction of Tat-regulated luciferase expression in TZM-bl cells [23,24]. ID50/ID80 represent the serum dilutions that reduce relative luminescence units (RLU) by 50%/80% compared to virus controls (after subtracting background). Two Env-pseudotyped viruses were used—a Tier 1A subtype C (MW965.26) and a Tier 2 vaccine strain (BG505/T332N)—with a SVA-MLV negative control to monitor non-specific effects.

#### 2.4.3. Infected cell antibody-binding assay (ICABA)

The assay quantified serum antibody binding to HIV-1 IMC BG505–infected CEM.CCR5.NKR cells versus mock-infected cells (to assess background) [25]. Participant sera (1:100 dilution) and controls—positive (mAb mix) and negative (Synagis mAb)—were incubated with the target cells, and antibody binding was measured by flow cytometry. Each sample was run in duplicate on separate plates for mock and infected cells.

#### 2.4.4. GranToxiLux antibody-dependent cell-mediated cytotoxicity (ADCC-GTL)

ADCC activity was quantified by net granzyme B (GTL) uptake, calculated as the percentage of GTL-positive target cells in the presence of serum minus that in target/effector wells lacking antibody [26].

#### 2.4.5. ADCC Luciferase

A modified luciferase-based ADCC assay employed HIV-1 IMC BG505–infected CEM.NKRCCR5 cells as targets and PBMCs from an HIV-seronegative donor (effector:target ratio of 30:1) [25,27]. Following a 6-h co-culture at 37 ^◦^C (with serial serum dilutions starting at 1:50), luminescence from remaining intact target cells was measured. Percent specific killing was calculated as: 100 × [(RLU in target+effector wells – RLU in test wells) / (RLU in target+effector wells)].

#### 2.4.6. B Cell Phenotyping

HIV-1 Env–specific B cells were characterized from PBMCs using fluorescent tetramers made by conjugating biotinylated Trimer AB05 gp140 and Trimer 4571 to SA-PE and SA-AF647. Non-B cells were excluded using markers CD3, CD14, and CD56. B cells were identified by CD19 and CD20 expression; memory B cells were defined as CD19 + CD20+ lacking IgD,

#### 2.4.7. Electron microscopy-based polyclonal epitope mapping (EMPEM)

Complexes of polyclonal Fab with Trimer 4571 were diluted to 0.03 mg/mL and adsorbed onto carbon-coated electron microscopy (EM) grids. The grids were stained with 2% uranyl formate for 45 s and imaged using a TFS Glacios TEM equipped with a TFS Falcon IV detector (200 keV, 73,000× magnification, 1.89 Å/pixel). Data collection was automated using EPU Multigrid (TFS) and processed using Relion 4.0 [28]. Following three rounds of 2D classification, particles from classes corresponding to immune complexes were subjected to 3D refinement with C3 symmetry and a 40 Å low-pass filtered map of HIV Env ectodomain as the initial model (based on PDB 6V0R). Following 3D refinement, C3 symmetry expansion was applied to the particles and 7 separate focused 3D classification “skip align” jobs were run (K = 10), each with a 40 Å diameter spherical mask over key HIV Env epitopes. The names of the epitopes and reference structures used for orienting the masks are: 1) gp41-base (PDB 6X9R), 2) gp41-GH (PDB 7L8U), 3) gp41-FP (PDB 7L8T), 4) gp120-GH (PDB 7L8B), 5) C3V5 (PDB 7L86), 6) CD4bs and gp120 interface (PDB 7L8X), and 7) V1V2V3 (PDB 7L8E). For each epitope 3D classification, classes with visible Fab density were selected and subjected to 3D refinement, 2D classification, and a second round of 3D classification. This was repeated until the reconstruction improved or no change was noted. Final reconstructions were visually inspected and assigned the correct epitope label. Visualization and image generation was performed using UCSF ChimeraX [29]. Representative EM maps have been deposited to the Electron Microscopy Data Bank. The assay is further described in [13,30,31].

#### 2.4.8. Intracellular Cytokine Staining (ICS) Assay

A validated 27-color ICS flow cytometry assay was used to evaluate HIV-1–specific CD4+ and CD8+ T cell responses as previously described (Table S2 and Fig. S7) [32–34]. Overlapping 15-mer peptides (11-amino acid overlap) representing BG505 SOSIP.664 gp140 were divided into two pools—BG505 gp120 (128 peptides) and gp41 (37 peptides). Similarly, peptides corresponding to the AB05 envelope (22 peptides for gp120 and 13 for gp41) were synthesized and pooled; the BG505 and AB05 pools were combined into BG505 AB05 gp120 and gp41 pools. Each peptide was used at a final concentration of 1 μg/mL. Overall Env-specific responses were defined as the sum of responses to both pools, with positivity determined by reactivity in at least one pool. Primary analyses focused on IFN-γ and/or IL-2 expression for overall T cell responses, IL-4/IL-5/IL-13 (with CD154 co-expression) for CD4+ Th2 responses, and IL-21 in CD4+ cells.

### 2.5. Statistical analyses

The sample size of the study was determined to provide reasonable precision in the assessment of the primary safety and immunogenicity endpoints. The randomization allocation sequence, done in blocks to ensure balance across arms, was obtained by computer-generated random numbers and provided to each clinical site through the HVTN Statistics and Data Management Center’s web-based randomization system. This was an open-label study for participants and site staff. Laboratory staff were blinded to group assignments, whenever feasible.

**Fig. 1.**
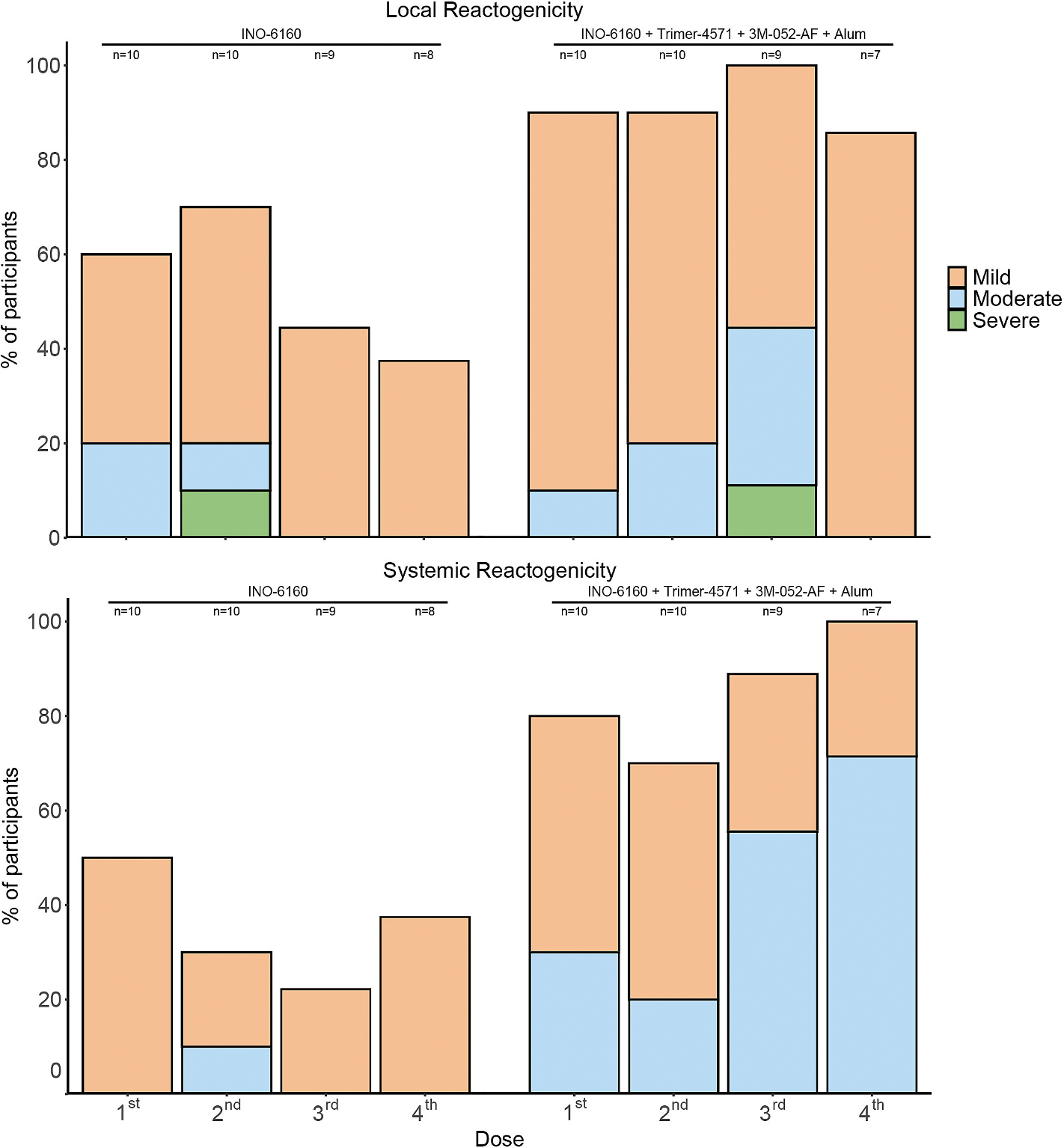
Solicited (“Reactogenicity”) Adverse Events in HVTN 304 participants. Reactogenicity was classified as mild, moderate, or severe. Percent of participants experiencing reactogenicity per group per dose is reported.

All randomized participants were included in the safety analyses. The number and percentage of participants experiencing each adverse event or reactogenicity symptom were tabulated by severity and vaccination regimen. All randomized participants with reliable assay data based on blood draw dates within the allowable visit window were included in the immunogenicity analysis. Because of the low missing data rate (Fig. 1), complete-case analysis of the subset of participants with immunogenicity data was used and is expected to give similar answers as methods that would impute missing values.

For the ICS assay, positivity for a peptide pool was based on comparing the percentage of T cells with positive staining for the cytokine of interest (e.g., IL-2 and/or IFN-γ) between the experimental and negative control wells using a one-sided Fisher’s exact test with a *p*-value cutoff for positivity of 10^−5^, and with a Bonferroni multiplicity adjustment for the number of peptide pools [35]. The same positivity criteria were also applied to the analysis of the B cell phenotyping assay comparing post-vaccination to pre-vaccination responses.

For the binding antibody assay, samples were declared to have positive responses if they met three conditions as previously described [36]. Base-specific binding antibody area under the curve (AUC) values for participants with a positive differential binding response were calculated by subtracting the AUC values of the mutant (BG505-MD39.3 Base-KO untagged) from the AUC values of the wildtype (BG505-MD39.3 untagged).

For the neutralization antibody assay, response to a virus/isolate was considered positive if the neutralization titer was above 10 (the lowest dilution tested).

Two-sided 95% confidence intervals (CI) for binomial proportions were calculated using the Wilson score method [37]. Wilcoxon rank sum tests were used to compare the response magnitudes between groups at a given time-point. Statistical significance for these comparisons between the two treatment groups is indicated in each figure.

All figures were created in R version 4.04 [38], and all safety data tabulations were performed in SAS Version 9.4 [39].

## 3. Results

### 3.1. HVTN 304 vaccine regimen was safe and generally well-tolerated

Twenty participants were enrolled: 10 in Group 1 (DNA-only) and 10 in Group 2 (DNA + protein) (Fig. S1, Table S1). DNA vaccination with INO-6160 delivered via ID EP was safe and well tolerated, either alone or in combination with an adjuvanted protein vaccine delivered IM. There were no serious adverse events or adverse events of special interest in any participant, and no unsolicited AEs deemed related to study product in any participant. Unsolicited AEs deemed unrelated to study product were generally consistent with other phase I trials in healthy adults (Fig. 1 and Supplementary File 1).

The tolerability profile of the vaccine regimen (as assessed by solicited adverse events) differed by group. For local solicited adverse events, more participants experienced mild to moderate local injection site solicited adverse events (“reactogenicity”) with the addition of the Env protein at the third and fourth dose as compared to the third and fourth dose of the DNA vaccine alone. Importantly, no participant reported severe pain. Erythema and induration were generally similar across the two groups, with most participants reporting some mild to moderate erythema and one participant in Group 1 and one participant in Group 2 reporting severe erythema.

Consistent with previous experience with 3M-052-AF adjuvanted vaccines, solicited systemic adverse events (“reactogenicity”) were more frequently reported in the group receiving the adjuvanted protein dose when compared to the DNA-only group. For example, ~50% of participants reported systemic AEs of moderate severity after receipt of the protein/DNA coadministration at dose 3 and dose 4, whereas 0/10 reported moderate-or-greater severity systemic AEs after the third dose of DNA only (Fig. 1 and Supplementary File 1).

One out of 9 participants (11%) in Group 1 discontinued further vaccinations due to local reactogenicity. Three out of 10 participants (30%) in Group 2 discontinued, two due to reactogenicity and one due to adverse events related to the EP delivery (Fig. S1).

### 3.2. Immunogenicity assessments

Humoral and cellular immunogenicity were assessed at 2 weeks following the second, third, and fourth immunizations. It is important to note that the treatment regimens were identical for the two treatment groups through the first two immunizations, and therefore, immunogenicity is expected to be similar until the third and fourth immunizations when Group 2 also received coadministration with the Trimer 4571 adjuvanted with 3M-052/Alum delivered IM.

#### 3.2.1. Env-specific binding antibodies were mostly directed to the Trimer base

**Fig. 2.**
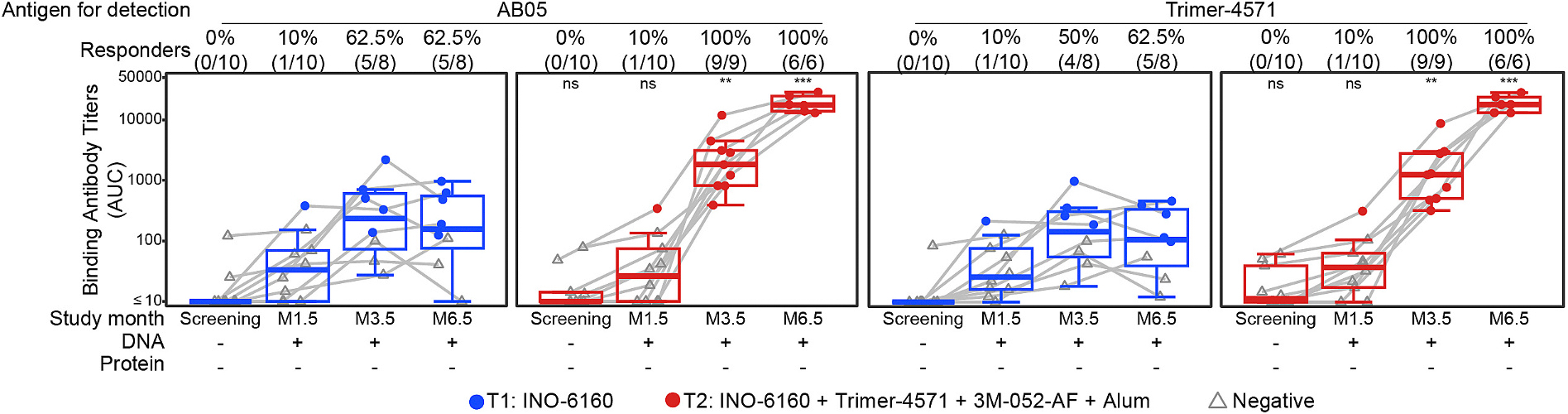
Vaccine-specific binding antibody responses against AB05 and Trimer 4571 at month 1.5 (M1.5), month 3.5 (M3.5) and month 6.5 (M6.5), two weeks after the second, third and fourth vaccinations, respectively. The frequency and magnitude of IgG binding antibodies specific for the AB05 trimer encoded by the DNA and the Trimer 4571 protein boost were measured by BAMA. Response magnitude to each antigen for detection is shown as area under the curve (AUC). Single dilution of 1:50. Responders to each antigen for detection are shown as % (N/total) at each study month. Positive responses are shown in filled circles; negative responses are shown in open gray triangles. Box plots represent the distribution for all participants (the upper and lower quartiles and the median). ns = not significant; ** indicates *p* ≤ 0.01; *** indicates *p* ≤ 0.001.

**Fig. 3.**
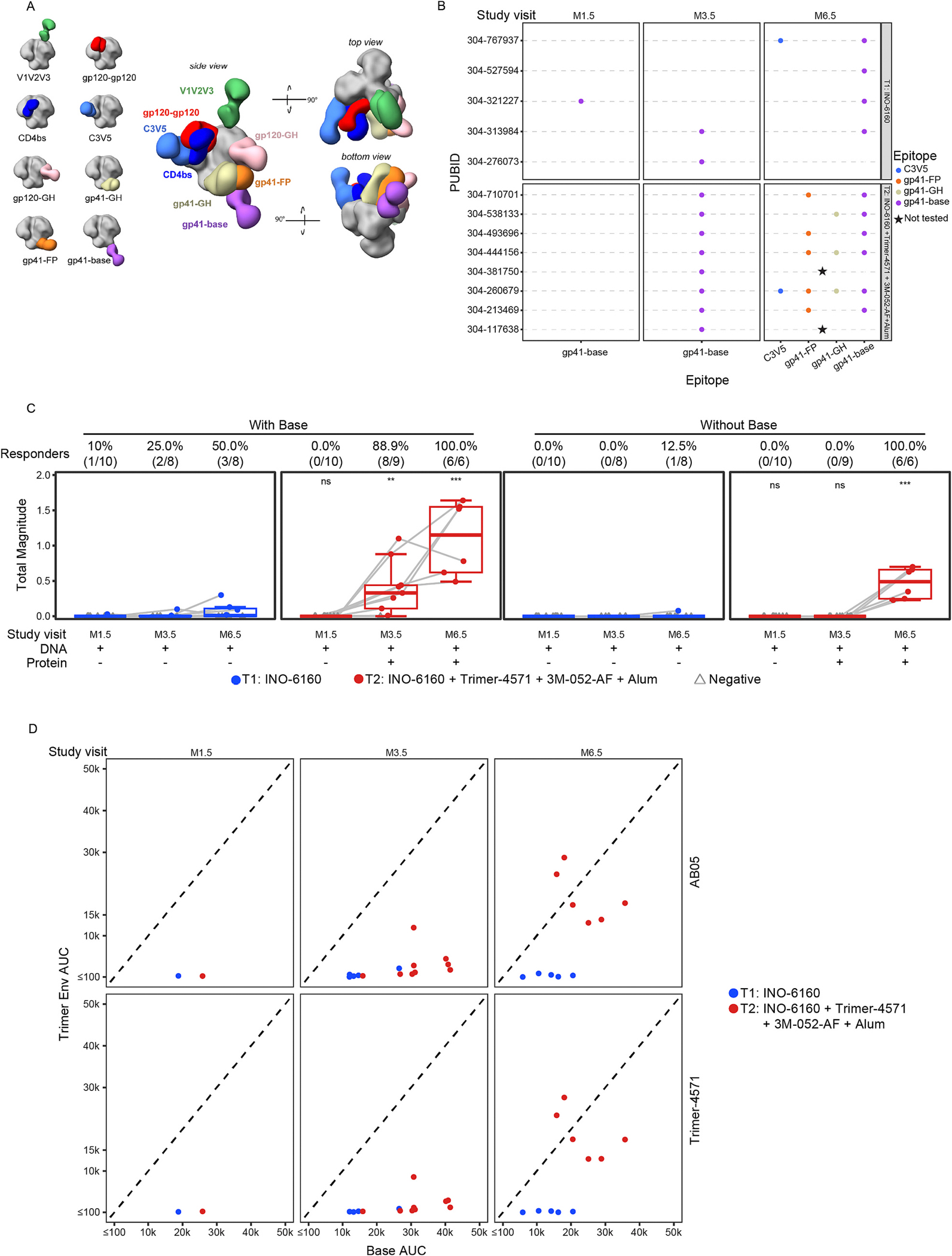
Antibody binding to non-physiological base of Env trimer at month 1.5 (M1.5), month 3.5 (M3.5), and month 6.5 (M6.5). (A) Schematic of epitope (gp41 base, gp41 fusion peptide (FP), gp41 glycan hole (GH) and C3V5) binding to Env trimer visualized by Electron Microscopy Polyclonal Epitope Mapping (EMPEM) direct semiquantitation. (B) A participant-level plot showing which epitopes had detectable antibody binding at each study month by treatment group. (C) The number of final particles belonging to each epitope was divided by the total number of particles in the initial (C3) 3D refinement. This value, which can range from 0 to 3 due to C3 symmetry expanded particles used in the 3D classification steps, is described as the *EMPEM magnitude*. Response is shown as total magnitude summing the positive responses of each epitope (gp41 base, gp41 fusion peptide (FP), gp41 glycan hole (GH) and C3V5, left) compared to the total but excluding the gp41-base (right). Positive responders are reported as % (N/total) for each study month. Positive responses are shown in filled circles; negative responses are shown in open gray triangles. Box plots represent the distribution for all participants (the upper and lower quartiles and the median). ns = not significant; ** indicates p ≤ 0.01; *** indicates p ≤ 0.001. (D) Base-specific IgG binding antibodies are determined by BAMA as the difference in the area under the curve (AUC) between a wildtype BG505 MD39.3 trimer and a base knockout (KO) trimer labeled as Base AUC on the x-axis vs. the two vaccine Trimer Env AUC on the y-axes. The dotted line shows the line of identity. Data points are color-coded by treatment group.

Env-specific binding antibodies to either the AB05 DNA encoded antigen Env Trimer or the recombinant soluble Env trimer Trimer 4571 were only detected in one participant in each of the groups after the second DNA vaccination (Month 1.5, Fig. 2). After the third DNA vaccination (Month 3.5), 62.5% of the participants had these responses which were not boosted by the fourth DNA vaccination in Group 1. All participants in Group 2 that received Trimer 4571 had binding antibody responses after the third and fourth vaccinations, and the magnitude of the responses was boosted by the fourth vaccination. These antibodies were primarily directed to the base of the Trimer as determined by the binding antibody assay and EMPEM (Fig. 3). Of the 6 participant samples that were tested at M6.5, 6 had responses to the gp41 base, 5 had responses to gp41 fusion peptide (FP), 3 had responses to gp41 glycan hole (GH), and 1 had a response to C3V5 (Fig. 3A and Fig. 3B). Differential binding to a base knock-out Env reveals dominant base binding for both groups at Month 3.5, with substantial non-base binding only for Group 2 at Month 6.5 (Fig. 3C). EMPEM further revealed that only after receiving the second dose of Trimer were there responses directed to other Env epitopes, although at lower magnitudes than the base-directed responses (Fig. 3D).

#### 3.2.2. Neutralizing antibodies were detected at low magnitudes

Autologous neutralizing antibodies measured using the BG505/T332N Env-pseudotyped vaccine strain virus were only detected in one Group 1 participant based on ID80 titers at baseline and month 3.5 (Fig. S2). Because this was detected at baseline and the magnitude was very low, this is likely a false-positive response. Neutralizing antibodies were detected to the Tier 1A virus subtype C strain MW965.26 in all group 2 participants after the second dose of Trimer 4571 (Fig. S2). Only very low-level responses were detected in a few Group 1 participants.

#### 3.2.3. ADCC activity was not detected for antibodies induced by DNA

ADCC was measured using complementary assays that measure different elements of antibody-mediated recognition and killing of infected cells. In the GranToxiLux assay, the ability of participant serum to mediate injection of cytolytic effector molecules into cells coated with a gp120 monomer is measured. As the target cells are coated with Env subtype A (BG505) gp120 monomeric protein, a detectable response suggests the presence of antibodies that should not be elicited if the trimer retains closed conformation (e.g. “off-target” responses). Using this assay, granzyme B activity was only detected in one participant in Group 2 at Month 6.5 (Fig. S3A).

Luciferase assay measures the ability of serum antibody to directly kill cells infected with an infectious molecular clone (IMC) matched to the virus in the presence of effector cells. Since an infectious molecular clone is used, antibodies to intact Env trimers could theoretically elicit these responses. Using this assay, specific killing (e.g. a reduction in luciferase) was also only detected after the administration of the recombinant protein Trimer 4571 (Group 2 at Month 6.5) in 3 of 6 participants (Fig. S3B).

A third assay related to ADCC detects antibodies binding to the surface of HIV-1-infected cells. Similar to the luciferase assay, binding antibodies were only detected in the group receiving trimer (Group 2 at month 3.5) that were then boosted with a second dose (5 of 6 participants at Month 6.5) (Fig. S3C). Thus, DNA-induced antibodies did not include detectable ADCC, although the possibility of priming these responses cannot be excluded.

#### 3.2.4. Vaccination led to an increase in T cell responses following third immunization

**Fig. 4.**
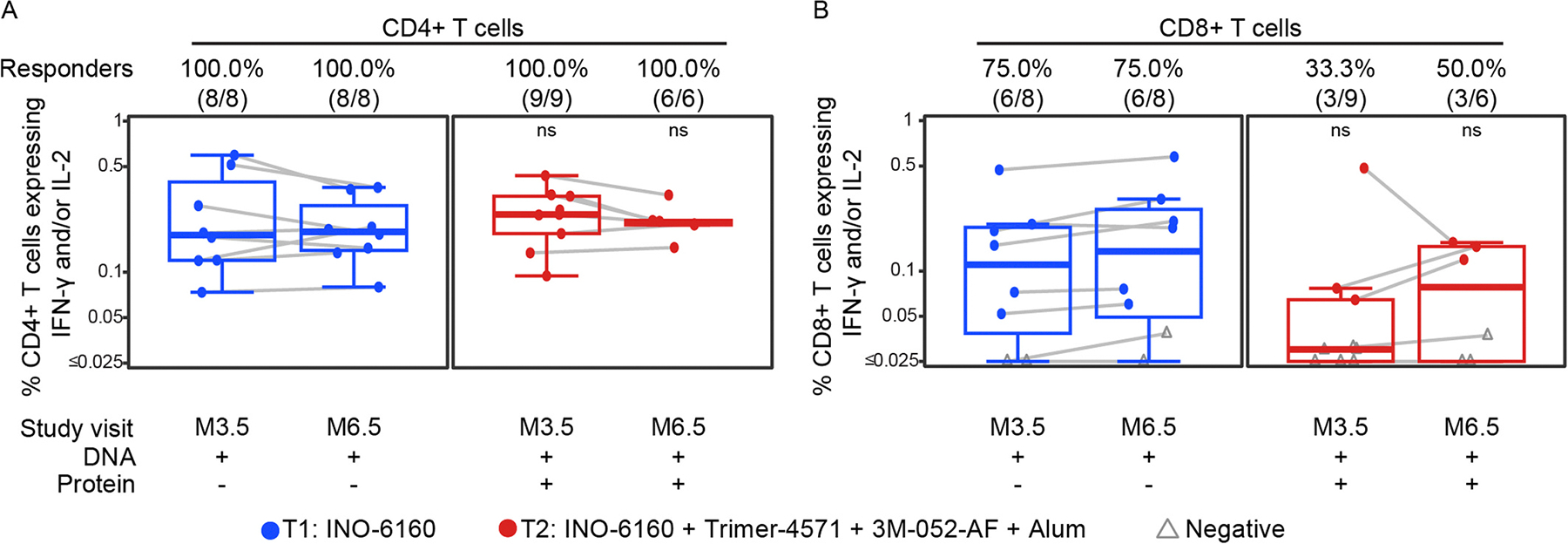
Vaccine-specific CD4þ and CD8þ T-cell responses at month 3.5 (M3.5) and month 6.5 (M6.5), two weeks after the third and fourth vaccinations, respectively. Env-specific responses were measured as the percent of CD4+ (A) or CD8+ (B) T cells expressing IFN-γ and/or IL-2 in response to gp120 and gp41 peptide pool stimulation (the sum is shown) and are background subtracted. Responders are reported as % (N/total) at each study month. Positive responses are shown in filled circles; negative responses are shown in open gray triangles. Box plots represent the distribution for all participants (the upper and lower quartiles and the median). ns = not significant.

Env-specific CD4+ T cells expressing IFN-γ and/or IL-2 were detected in all participants in both groups and there was no increase in the response with a fourth DNA vaccination vs. three DNA vaccinations in Group 1 (Fig. 4, S4, and S7). There was also no increase in response when Trimer 4571 was co-administered with DNA in Group 2 since the responses in the two groups were comparable. The magnitudes for these responses were as high as 0.6% of CD4+ T cells with medians between 0.18 and 0.24%. Env-specific CD8+ T cells expressing IFN-γ and/or IL-2 were detected in 75% of Group 1 participants at both post-third and post-fourth vaccination timepoints. Response rates were numerically lower (although not statistically significant) in Group 2 at 33% after the third and 50% after the fourth. Among the participants with positive responses, the magnitudes were similar to the CD4+ T cell responses, with a maximum of 0.58% and medians ranging between 0.08 and 0.2%. When separately analyzing responses to the gp120 and gp41 of Env, the gp120 seemed to induce a much higher CD4+ T cell magnitude response, although gp41-specific responses were detected in most participants (Fig. S5A). CD8+ T cell responses were primarily detected only for gp120 (Fig. S5B).

Env-specific CD4+ T cells expressing Th2 cytokines, IL-4, IL-5, or IL-13, were not detected. CD4+ T cells expressing IL-21 may identify circulating T follicular helper (Tfh) cells [40]. Env-specific CD4+ T cells expressing IL-21 were detected in 2 of 8 Group 1 participants and 4 of 9 Group 2 participants after the third vaccination (Fig. S6). These response magnitudes were low compared to the IFN-γ and/or IL-2 responses and are near the detection limit of the assay and the pattern of IL-21 response was not markedly different between gp41 and gp120 peptides (Fig. S6). There were other low-level responses after the third vaccination that were not determined to be positive and that decreased by the fourth vaccination, perhaps indicative of responses below detection. In general, most participants had evidence of circulating Tfh cells after the third vaccination that were not present after the fourth vaccination.

#### 3.2.5. Env-specific B cell responses were robust in Group 2 participants

**Fig. 5.**
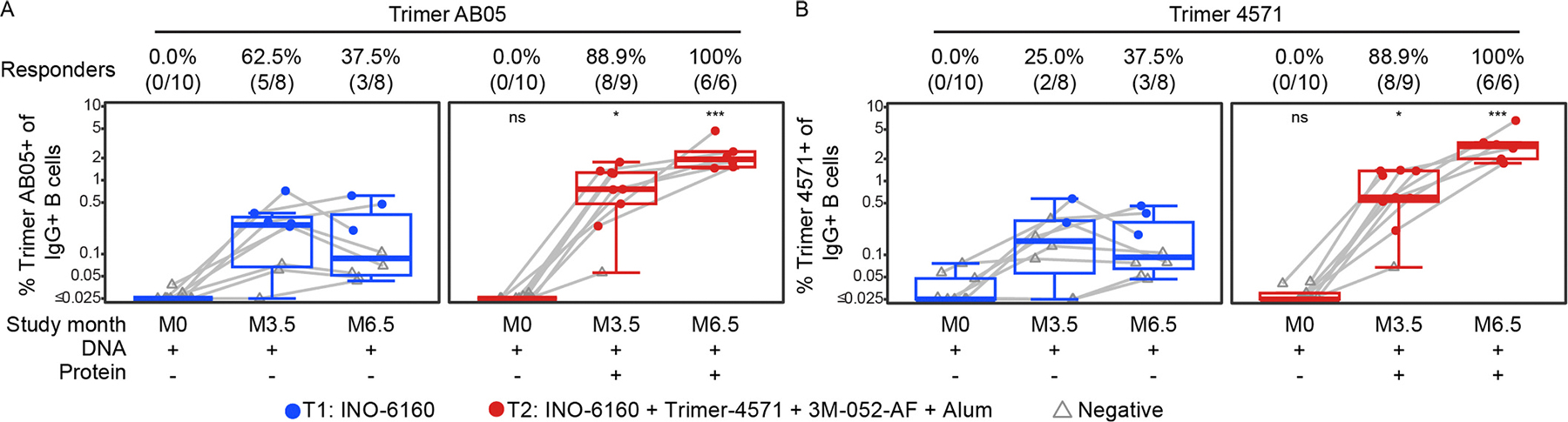
Vaccine-specific B-cell responses against AB05 and Trimer 4571 at month 0 (M0), month 3.5 (M3.5) and month 6.5 (M6.5), baseline pre-vaccination, two weeks after the third and fourth vaccinations, respectively. Responses were measured as the percent of IgG+ B cells staining for either Trimer AB05 (A) or Trimer 4571 (B) fluorescent probes. Responders are shown as % (N/total) for each study month. Positive responses are shown in filled circles; negative responses are shown in open gray triangles. Box plots represent the distribution for all participants (the upper and lower quartiles and the median). ns = not significant; * indicates *p* ≤ 0.05; *** indicates *p* ≤ 0.0001.

Fluorescent-labeled probes for the envelope proteins encoded in the DNA (referred to as Trimer AB05) and the Trimer 4571 were used to measure the proportion of memory B cells binding to these probes. At month 3.5, 8 of 9 participants in Group 2 had detectable Env-specific IgG+ B cells for either probe, and this increased to 100% after the fourth vaccination (Fig. 5 and S8). A smaller proportion in Group 1 had these responses at M3.5 (5 of 8 for Trimer AB05, and 2 of 8 for Trimer 4571) and was largely not boosted after a fourth dose of the DNA.

## 4. Discussion

Delivery of stabilized HIV Env trimer DNA by electroporation of DNA plasmids and host transcription/translation represents a potentially attractive vaccine modality for HIV vaccine strategies intended to elicit broadly neutralizing antibody responses. Consistent with preclinical data, Env induced by DNA in this study elicited Env-generated B cells capable of recognizing intact trimer. The results demonstrate proof of principle that intact recombinant HIV Env can be encoded by DNA plasmids and can elicit a memory B cell response capable of binding the intact trimer. Additionally, the ID EP delivery of the HIV Env DNA was safe, as no serious AEs were observed in any participant, and tolerability was comparable to other licensed vaccine modalities, such as mRNA. The study demonstrates that ID EP DNA vaccination via adaptive electroporation therefore represents a potential method for delivering structurally intact HIV immunogens.

Despite inducing stabilized HIV Env trimer, the regimen did not elicit any broadly neutralizing antibodies in serum, and the majority of the immune response was directed at the immunodominant base. This is consistent with other methods of delivering or inducing native-like HIV Env trimers by vaccination [13,16,36]. Our findings demonstrate that induction of Env trimers by DNA has many of the same drawbacks as other vaccine modalities tested in HIV vaccine clinical trials to date, such as immunodominant response to the base of the trimer. Additionally, unlike other modalities of Env delivery, DNA alone was incapable of eliciting any detectable ADCC activity with this immunogen. Whether this was due to overall low titers or an intrinsic property of DNA priming should be explored in future studies; we would note that we did observe detectable ADCC with recombinant protein. Future designs could refine the immunogen to reduce base binding and focus the response on specific sites which may enhance other functional activities such as ADCC.

The antibody responses elicited with DNA-only vaccination in HVTN 304 somewhat differ from those observed in the ID/EP HVTN 098 study [4]. In HVTN 098, DNA vaccination with a monomer resulted in durable antibody response in almost all participants (>90% for a vaccine-matched gp140 antigen six months after vaccination). Binding antibodies against the vaccine matched trimer in the DNA-only group were detected in only 3/8 participants two weeks after the third dose in the current study. In HVTN098, both intradermal and intramuscular methods were explored and there were minimal differences in the immunogenicity between the two approaches (and the IM approach was much less well tolerated), so we do not believe that an IM route would have made a substantial difference. Similar to the protein group, most of these antibodies were directed against the immunodominant “base” of the trimer. For context, after two doses of a protein adjuvanted with 3M-052, a dilution of 1:156250 was required to avoid issues with saturation in the binding antibody readout [13]; the DNA arm of the present trial had linear readings with a dilution of 1:50 (e.g. ~3–4 logs lower binding antibody). Similar to previous observations, DNA priming followed by a protein boost led to greater antibody responses compared with DNA boosting alone.

A complete absence of convincing Tier 1 neutralizing antibodies in the DNA only group is different than what was observed in HVTN 098 where Tier 1 neutralizing antibodies were detected in >50% of participants. This suggests that the DNA encoded Env trimer may have advantages for serological “on target” responses as compared to delivery of a monomer; Tier 1 neutralization is thought to be driven by internal epitopes. We did observe one participant with very weak neutralization titer at baseline that we would consider to be from some non-specific factor such as serum-induced cell toxicity.

The DNA vaccination elicited vaccine-specific T cells in 100% of group 1 participants [CD4 (8/8) and CD8 T cells (6/8)]. For group 2, there was no additive benefit on the CD4+ or CD8+ T cell response upon protein boosting. In fact, the CD8+ T cell response rate was numerically lower when the DNA was co-administered with the Trimer 4571 (although not statistically significant, likely due to a small sample size), suggesting a potential interference with MHC class 1 presentation of CD8 epitopes when protein is administered. This is surprising since both groups had received the same DNA vaccinations throughout the study, suggesting an effect of protein boost on DNA priming. In contrast, the CD4+ T cell response was unaffected by co-administration. In both groups, we observed a decrease in the circulating IL-21 producing CD4+ T cells after the fourth vaccination compared to the third. While we did not obtain LN samples, we would observe that circulating Tfh cells obtained from peripheral blood have dynamic expansion and contraction kinetics following vaccination. For example, the magnitude of vaccine circulating Tfh cells that produce IL-21 in the periphery is dynamic following vaccination with the neoantigen yellow fever with peak responses observed 14 days after vaccination [41]. With influenza vaccination, circulating Tfh cells experience substantial expansion and contraction in the periphery five, with peak five days and fluctuations that are more pronounced than other T cells subsets (e.g. Tregs) or Tfh cells isolated from the lymph node [42]. We would therefore speculate that the decline represents differences in the dynamics of the GC response but additional investigations would be required for firm conclusions. Prior evidence of competition was demonstrated in a clinical trial where a greater CD8+ T cell response was seen after administration of an adenovirus-vectored-vaccine encoding Gag and Pol compared to responses after a Gag/Pol/Env adeno vaccine with multiple HIV Envs [43]. Additionally, co-administration with Env impacted the quality of the cellular response, as polyfunctionality analysis revealed lower functionality scores for both CD4+ and CD8+ T cells for Gag and/or Pol, and reduced epitope breadth of Gag/Pol-specific T cell responses.

We conclude that DNA may be a very attractive modality for rapid iteration of vaccines based upon inducing CD8+ T cell responses (e.g. networked epitopes, or microconsensus, as in HVTN 098) [10,44]. In this regard, a recent ID/EP DNA vaccine encoding 40 neoantigens generated high CD8 T cell frequencies and clinical responses in an advanced hepatocellular carcinoma immunotherapy FIH study [45]. The flexibility of the DNA modality would potentially allow for evaluation of multiple CD8+ vaccine candidates, as it would have many of the manufacturing advantages of mRNA. mRNA vaccines using similar Env inserts did not elicit nearly as robust a CD8+T cell response [36]. Recent in vivo studies have demonstrated the stability of DNA-expressed stabilized trimer for several weeks [19,46]. It would be technically challenging to directly measure the structural integrity of the Env delivered by DNA in humans, but there are several indirect lines of evidence to suggest that at least some portion of the Env was structurally intact. These include a lack of reactivity to gp120 in the ADCC activity (although this could also reflect low overall titers), lack of significant Tier-1 neutralization activity (typically associated with “open” trimers and readily detected with DNA delivery of gp120 monomers [4], and an ability to provide a robust recall response with a recombinant trimer that is known to maintain structural integrity [16].

Previous investigations using monomeric Env derivatives have demonstrated that DNA priming has led to robust antibody responses with gp120 protein boosting compared to multiple protein-only administrations, and our study adds to this by suggesting that B cells are modestly primed without a great deal of serum antibody [18]. The mechanism behind the observation that the DNA modality primes a robust antibody response with protein is unclear, but it is plausibly related to the concept of antibody feedback, whereby the initial antibodies elicited by protein vaccines compete with the B cells for antigen [47]. Our observations that DNA alone induced a substantial B cell response but relatively low serum antibody levels might be a particularly attractive feature for the early stages of sequential germline targeting immunization strategies where a substantial B cell response is desired but not necessarily high serum antibody levels. We would caution that the overall magnitude of the B cell primed B cell response was lower than other that other modalities such as mRNA [36] or protein [13]; it is unlikely that antibody feedback alone explains the lower overall response as the other modalities elicited higher antibody titers, as well. There are a variety of plausible mechanisms including lower antigen exposure (compared to mRNA or protein), reduced innate cytokine stimulation, and altered antigen kinetics that should be explored in pre-clinical systems. In general, the ineffectiveness of repeated homologous vaccinations that drive immune responses higher is poorly understood [48].

Although we did not have a single-dose “protein only” arm for formal comparison, the antibody levels after the first dose of the BG505 derivative (at month 3.5) were comparable to two doses [13], suggesting that DNA primed a B cell response that could be recalled using a recombinant protein vaccine. Conceptually, a series of regimens targeting the nascent B cell response could be given quickly with DNA without the issue of competing antibodies; for the final step, high titers of durable bnAbs could be induced by other methods such as protein or nanoparticle vaccines. It is important to consider for trimer-based studies of DNA and perhaps other nucleic acid or viral vector-delivered HIV immunogens to compare soluble trimers with membrane-stabilized forms.

One of the strengths of this study is its robust, validated pipeline for immunologic readouts. Additionally, the study design allowed for a direct comparison of boosting the immune response to HIV Env with DNA versus with DNA plus a recombinant protein. Both methods of vaccine delivery (IM and ID EP) were safe and generally well tolerated. Potential weaknesses of the study include the small sample size and lack of placebo group, which limit the extent of analysis. For example, there was a numerical imbalance in sex-assigned at birth between groups one and 2 (4 versus 7, respectively), but the study is underpowered to investigate futher the influence of sex in the observed differences between the two groups. Meanwhile, cross-protocol analyses of baseline factors in multiple HIV vaccine trials combined have found female sex to be weakly associated with vaccine-induced immune responses (e.g., [49]). An additional limitation is the lack of a protein-only control; however, recombinant Env trimers have been delivered with various adjuvants in several other trials [13].

## 5. Conclusions

The current state of the HIV vaccine field is largely focused on the elicitation of broadly neutralizing antibodies via vaccination. Similar to other methods of delivery of soluble native-like HIV Env, the current regimen did not elicit bnAbs; thus, the development of other strategies that can achieve this desired response continues. Data from this trial suggest some novel and potentially useful features of DNA delivery of HIV Env trimers, as compared to recombinant protein, with a variety of adjuvants or other modalities such as mRNA. Specifically, the ability to induce a B cell response without the elicitation of a robust antibody response offers a potentially useful feature. Antibody-mediated inhibition of humoral responses has been observed in preclinical studies, clearly demonstrated in the presence of maternally transferred IgG, and more fully characterized in adults with COVID-19 vaccination [47,50,51]. If antibody feedback represents a challenge for sequential immunization strategies, DNA vaccines could represent an attractive option for the early steps in germline targeting sequential immunization strategies. The current trial represents an incremental step in the search for an effective HIV vaccine and suggests that DNA vaccination may contribute to this important goal.

## Supplementary Material

MMC1

MMC2

## Figures and Tables

**Table 1:** Study schema showing dosing schedule and route of administration per group. Eligible participants between 18 and 55 years of age, without HIV-1, in good overall health, and unlikely to acquire HIV during the study period were randomized into two groups. Each group received INO-6160 at Months 0, 1, 3, and 6. Participants in Group 2 received Trimer 4571/3M-052-AF/Alum at Months 3 and 6.

				Injection Schedule			
Group	N	Product/Dose	Route	Month 0	Month 1	Month 3	Month 6
1	10	INO-6160 / 2.0 mg	ID EP	X	X	X	X
2	10	INO-6160 / 2.0 mg	ID EP	X	X	X	X
		Trimer 4571 / 100 μg 3M-052-AF (5 μg) + Alum (500 μg)	IM			X	X
**Total**	20						

## Data Availability

De-identified data associated with this manuscript are available here: https://dataverse.harvard.edu/dataverse/hvtn304.

## Appendix A. Supplementary data

Supplementary data to this article can be found online at https://doi.org/10.1016/j.vaccine.2026.128487.
