# Supplementary material for: DNA electroporation of HIV Env elicits robust T cell responses and memory B cell responses with muted serum antibody levels that can be boosted with recombinant protein
Source: Vaccine. Author manuscript; Available in PMC 2026 May 21. (PMC13193312; doi:10.1016/j.vaccine.2026.128487)
Supplement: MMC1 [file NIHMS2159065-supplement-MMC1.docx]

**DNA electroporation of HIV Env elicits robust T cell responses and memory B cell responses with muted serum antibody levels that can be boosted with recombinant protein**

**Supplementary Materials**

**HVTN 304 Study Team:**

| Stephen C. De Rosa | Fred Hutchinson Cancer Center |
| --- | --- |
| Ronnie Matt Gravett | University of Alabama at Birmingham |
| William O. Hahn | Fred Hutchinson Cancer Center |
| Manuel Villaran | Fred Hutchinson Cancer Center |
| Yifan Zhu* | Fred Hutchinson Cancer Center |
| Yunda Huang | Fred Hutchinson Cancer Center |
| Lorel Schmitzberger | Fred Hutchinson Cancer Center |
| Laura Polakowski | NIAID |
| David Weiner | The Wistar Institute |
| Dan Kulp | The Wistar Institute |
| Mansi Purwar | The Wistar Institute |
| Laurent Humeau | Inovio Pharmaceuticals |
| Zachary Sagawa | AAHI |
| Lucio Gama | VRC |
| Jen Hanke | Fred Hutchinson Cancer Center |
| Amy Palin | NIAID |
| Shane Hebel | Boston, MA CAB |
| Bobby Christon-Walker | Birmingham, AL CAB |
| Gail Broder | Fred Hutchinson Cancer Center |
| Rafael Gonzalez | Fred Hutchinson Cancer Center |
| Jorge Benitez | Columbia Research Unit |
| Megan Jones | Fred Hutchinson Cancer Center |
| Carrie Sopher | Fred Hutchinson Cancer Center |
| Liz Briesemeister | Fred Hutchinson Cancer Center |
| Sophie Arogbonlo | Fred Hutchinson Cancer Center |
| Brian Ingersoll | Fred Hutchinson Cancer Center |
| April Randhawa | Fred Hutchinson Cancer Center |
| Sujata Vijh | NIAID |
| Meg Trahey | Fred Hutchinson Cancer Center |
| Smitha Sripathy | Fred Hutchinson Cancer Center |
| Rochelle Rogers | NIAID |
| Kelly Colsh | NIAID |
| Anders McConachie | Fred Hutchinson Cancer Center |

***** replaced by Yunda Huang

**Figure S1: HVTN 304 CONSORT diagram showing participant flow from enrollment to sample analysis.** N=20 participants were enrolled and randomized into two groups of N=10. Participants were followed through Follow-Up and Analysis. G: Grade


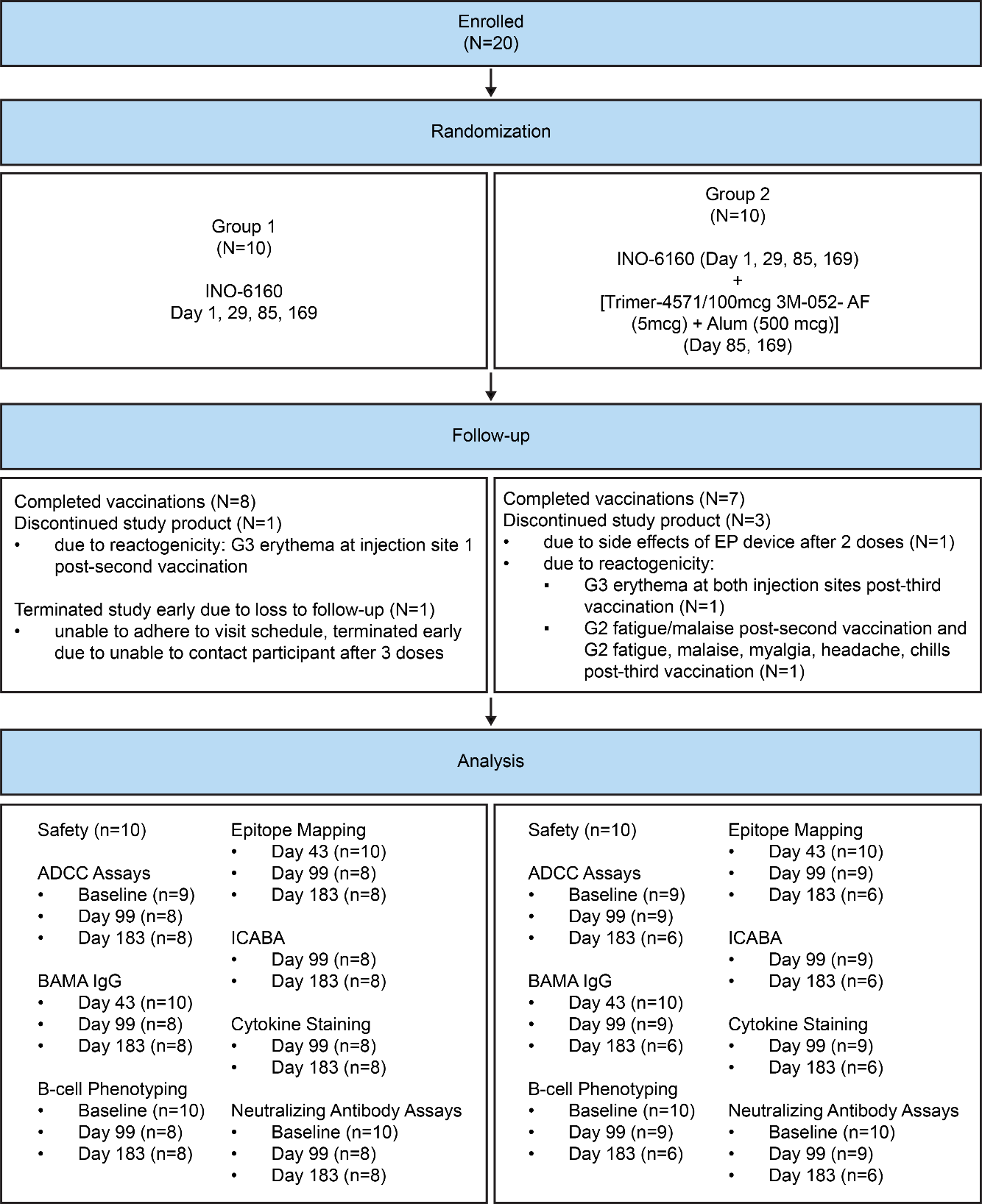


**­Table S1: Participant demographics in HVTN 304.**

|  | **Total** | **INO-6160 / 2.0 mg** | **INO-6160 / 2.0 mg**  **Trimer-4571 / 100 mcg 3M-052-AF (5 mcg) + Alum (500 mcg)** |
| --- | --- | --- | --- |
| **Total Enrolled (N)** | 20 | 10 | 10 |
| **Assigned Sex at Birth** |  |  |  |
| Male | 11 (55.0%) | 7 (70.0%) | 4 (40.0%) |
| Female | 9 (45.0%) | 3 (30.0%) | 6 (60.0%) |
| **Gender** |  |  |  |
| Male | 11 (55.0%) | 7 (70.0%) | 4 (40.0%) |
| Female | 8 (40.0%) | 3 (30.0%) | 5 (50.0%) |
| Transgender male | 0 (0.00%) | 0 (0.00%) | 0 (0.00%) |
| Transgender female | 0 (0.00%) | 0 (0.00%) | 0 (0.00%) |
| Gender queer | 0 (0.00%) | 0 (0.00%) | 0 (0.00%) |
| Gender variant or gender non-conforming | 1 (5.0%) | 0 (0.00%) | 1 (10.0%) |
| Another self-identified gender | 0 (0.00%) | 0 (0.00%) | 0 (0.00%) |
| Prefer not to answer | 0 (0.00%) | 0 (0.00%) | 0 (0.00%) |
| **Ethnicity** |  |  |  |
| Hispanic or Latino | 2 (20.0%) | 1 (10.0%) | 1 (10.0%) |
| Not Hispanic or Latino | 18 (80.0%) | 9 (90.0%) | 9 (90.0%) |
| **Age (years)** |  |  |  |
| Less than 18 | 0 (0.00%) | 0 (0.00%) | 0 (0.00%) |
| 18 - 20 | 1 (5.0%) | 0 (0.00%) | 1 (10.0%) |
| 21 - 30 | 7 (35.0%) | 3 (30.0%) | 4 (40.0%) |
| 31 - 40 | 10 (50.0%) | 6 (60.0%) | 4 (40.0%) |
| 41 - 50 | 2 (10.0%) | 1 (10.0%) | 1 (10.0%) |
| 51 - 55 | 0 (0.00%) | 0 (0.00%) | 0 (0.00%) |
| Median (range) | 33 (19-45) | 34 (24-42) | 30.5 (19-45) |
| **Race** |  |  |  |
| White | 16 (80.0%) | 9 (90.0%) | 7 (70.0%) |
| Black or African American | 0 (0.00%) | 0 (0.00%) | 0 (0.00%) |
| Asian | 1 (5.0%) | 1 (10.0%) | 0 (0.00%) |
| Native Hawaiian or other Pacific Islander | 0 (0.00%) | 0 (0.00%) | 0 (0.00%) |
| American Indian or Alaska Native | 0 (0.00%) | 0 (0.00%) | 0 (0.00%) |
| Multi-racial | 3 (15.0%) | 0 (0.00%) | 3 (30.0%) |
| Other | 0 (0.00%) | 0 (0.00%) | 0 (0.00%) |
| **SPA Frequencies** |  |  |  |
| V2.0 – Enrollment | 20 (100.0%) | 10 (100.0%) | 10 (100.0%) |
| V4.0 – Day 29 | 20 (100.0%) | 10 (100.0%) | 10 (100.0%) |
| V5.0 – Day 85 | 18 (90.0%) | 9 (90.0%) | 9 (90.0%) |
| V6.0 – Day 169 | 15 (75.0%) | 8 (80.0%) | 7 (70.0%) |

**Figure S2: Vaccine-specific ID80 neutralizing antibody responses at baseline, month 3.5 (M3.5) and month 6.5 (M6.5), two weeks after the second, third and fourth vaccinations, respectively.** The upper panel shows responses for an Envelope-pseudotyped virus (BG505/T332N) similar to the vaccine strains that exhibits a tier 2 neutralization phenotype. The lower panel shows responses to a tier 1A subtype C virus, MW965.26. Responses were measured as titer. Responders are reported as % (N/total) at each study month. Positive responses are shown in filled circles; negative responses are shown in open gray triangles. Box plots represent the distribution for all participants (the upper and lower quartiles and the median). ns=not significant; *** indicates p≤0.001


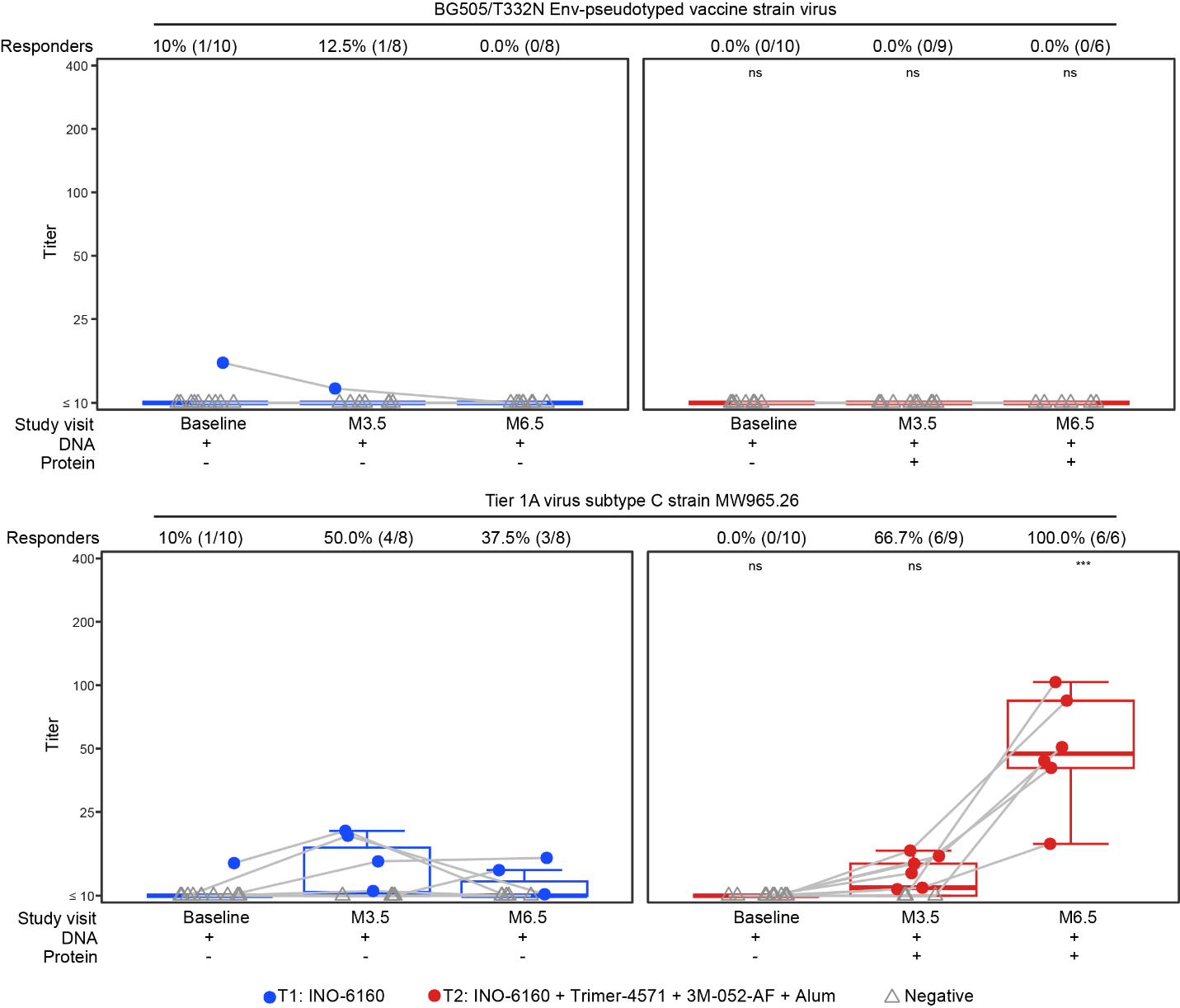


**Figure S3: Antibody-dependent cell-mediated cytotoxicity (ADCC).** (A) ADCC was quantified as net granzyme B activity (difference in the percent of target cells positive for GTL when effector cells are incubated in the presence or absence of antibodies from serum samples). A positive response was defined as peak activity greater than or equal to 8%. Responders are reported as % (N/total) per study month in each group. (B) Reduction in luciferase activity is shown as % specific killing per study month. A response is defined as positive if the peak baseline-subtracted % loss luciferase activity is greater than or equal to 10% for either the 1:50 or 1:200 dilution. Responders are reported as % (N/total) per study month in each group. (C) Percent of HIV-1 infectious molecular clone BG505-infected CEM.CCR5.NKR cells that bind to serum antibodies. Responses are considered positive if mock and baseline subtracted %IgG+ is greater than 5% at dilution 1:100. Responders are reported as % (N/total) per study month in each group. Positive responses are shown in filled circles; negative responses are shown in open gray triangles. Box plots represent the distribution for all participants (the upper and lower quartiles and the median). ns=not significant; ** indicates p≤0.01


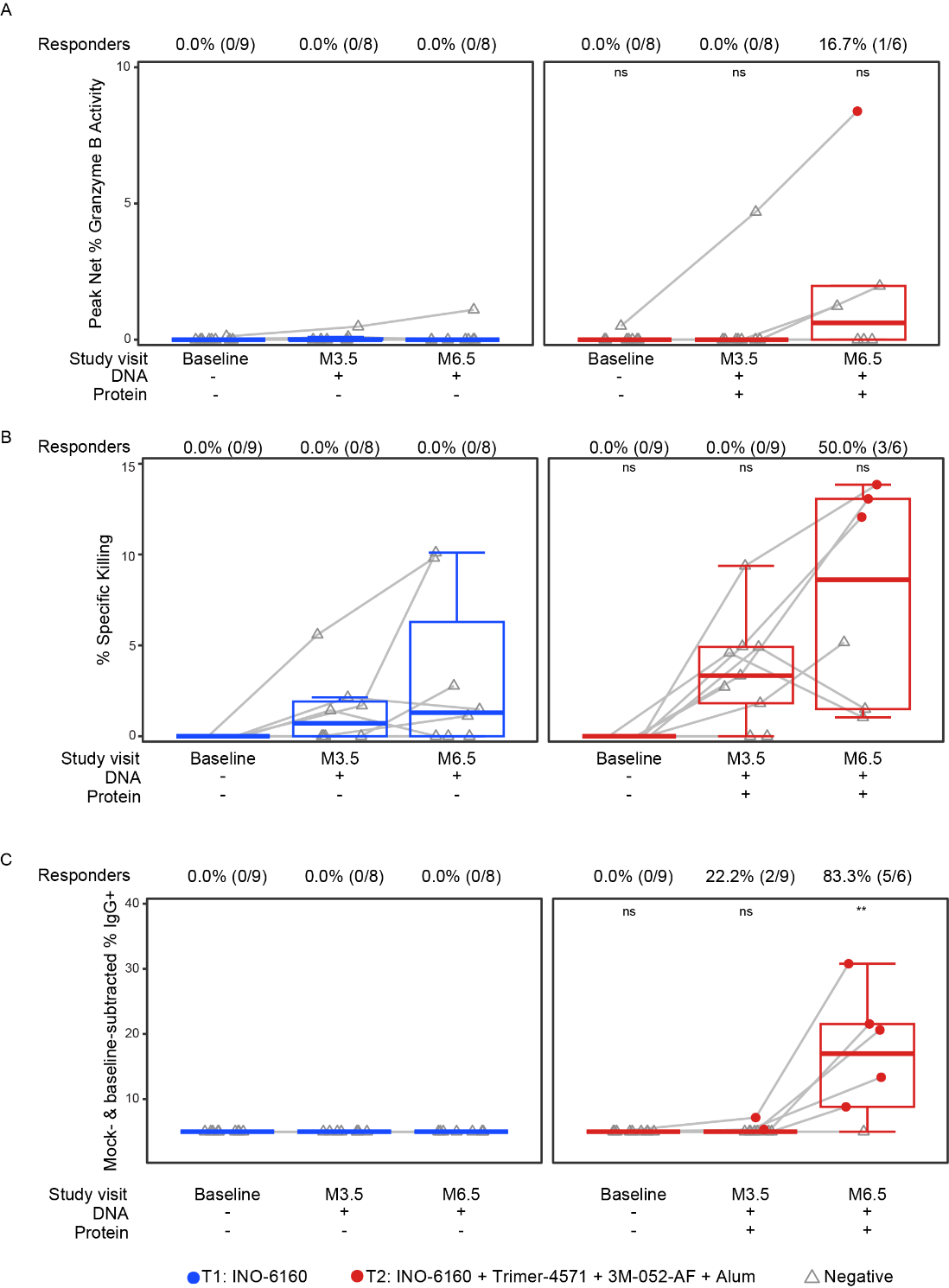


**Figure S4: Sex-stratified vaccine-specific CD4+ and CD8+ T-cell responses at month 3.5 (M3.5) and month 6.5 (M6.5), two weeks after the third and fourth vaccinations, respectively.** Env-specific Responses were measured as the percent of CD4+ or CD8+ T cells expressing IFN-γ and/or IL-2 in response to gp120 and gp41 peptide pool stimulation (the sum is shown) and are background subtracted. Responders are reported as % (N/total) at each study month. Female positive responses are shown in filled circles; male positive responders are shown as filled triangles. Female non-responders are shown in open circles; male non-responders are shown in open triangles. Box plots represent the distribution for all participants (the upper and lower quartiles and the median).**
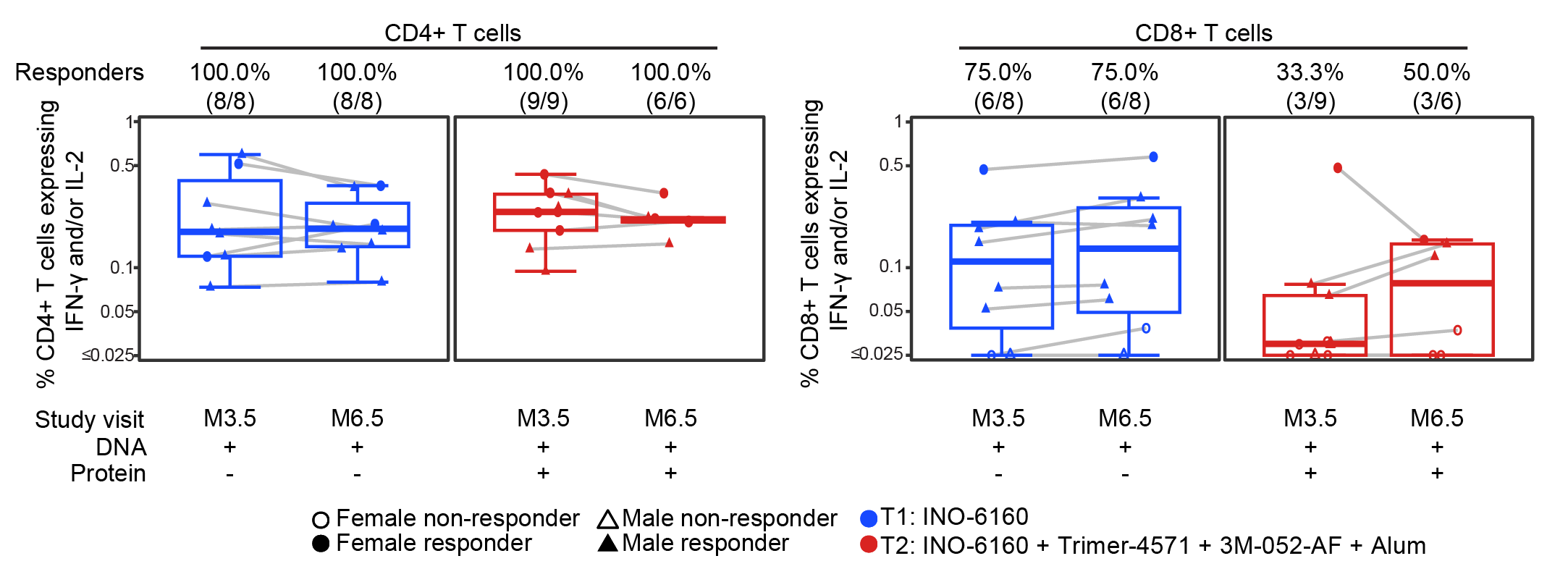
**

**Figure S5: Env-specific T cell response.** CD4+ (A) and CD8+ (B) T cells expressing IFN-γ and/or IL-2 in response to the BG505 AB05 gp41 (left) and gp120 peptide pools (right) at M3.5 and M6.5 and are background subtracted. Responders are reported as % (N/total). Positive responses are shown in filled circles; negative responses are shown in open gray triangles. Box plots represent the distribution for all participants (the upper and lower quartiles and the median). ns=not significant


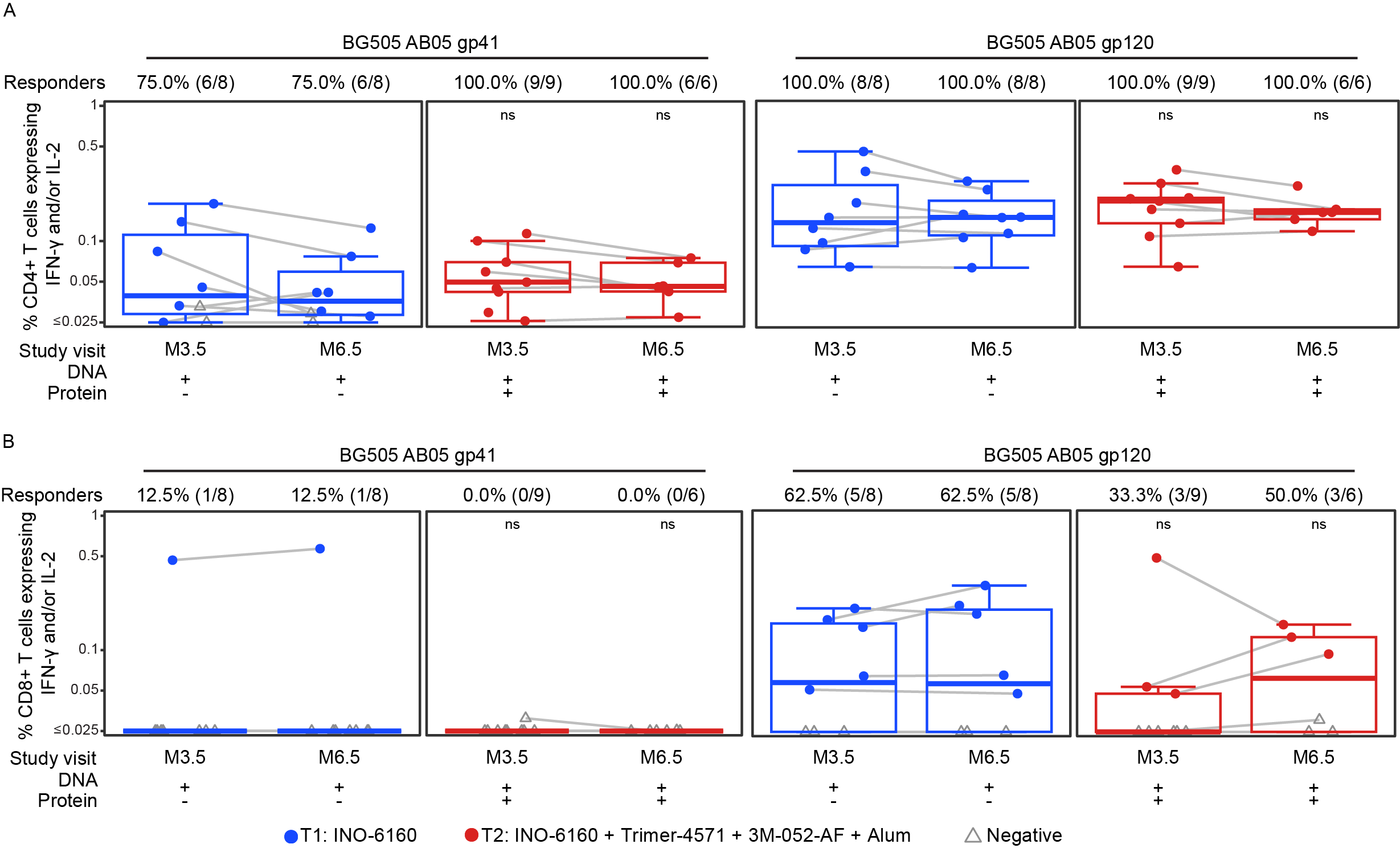


**Figure S6 T cell responses to total Env.** Percent of CD4+ T cells expressing IL-21 in response to the (A) Total, (B) gp120, and (C) gp41 peptide pool stimulations at M3.5 and M6.5 per treatment groups. Responders are reported as % (N/total). Positive responses are shown in filled circles; negative responses are shown in open gray triangles. Box plots represent the distribution for all participants (the upper and lower quartiles and the median). ns=not significant


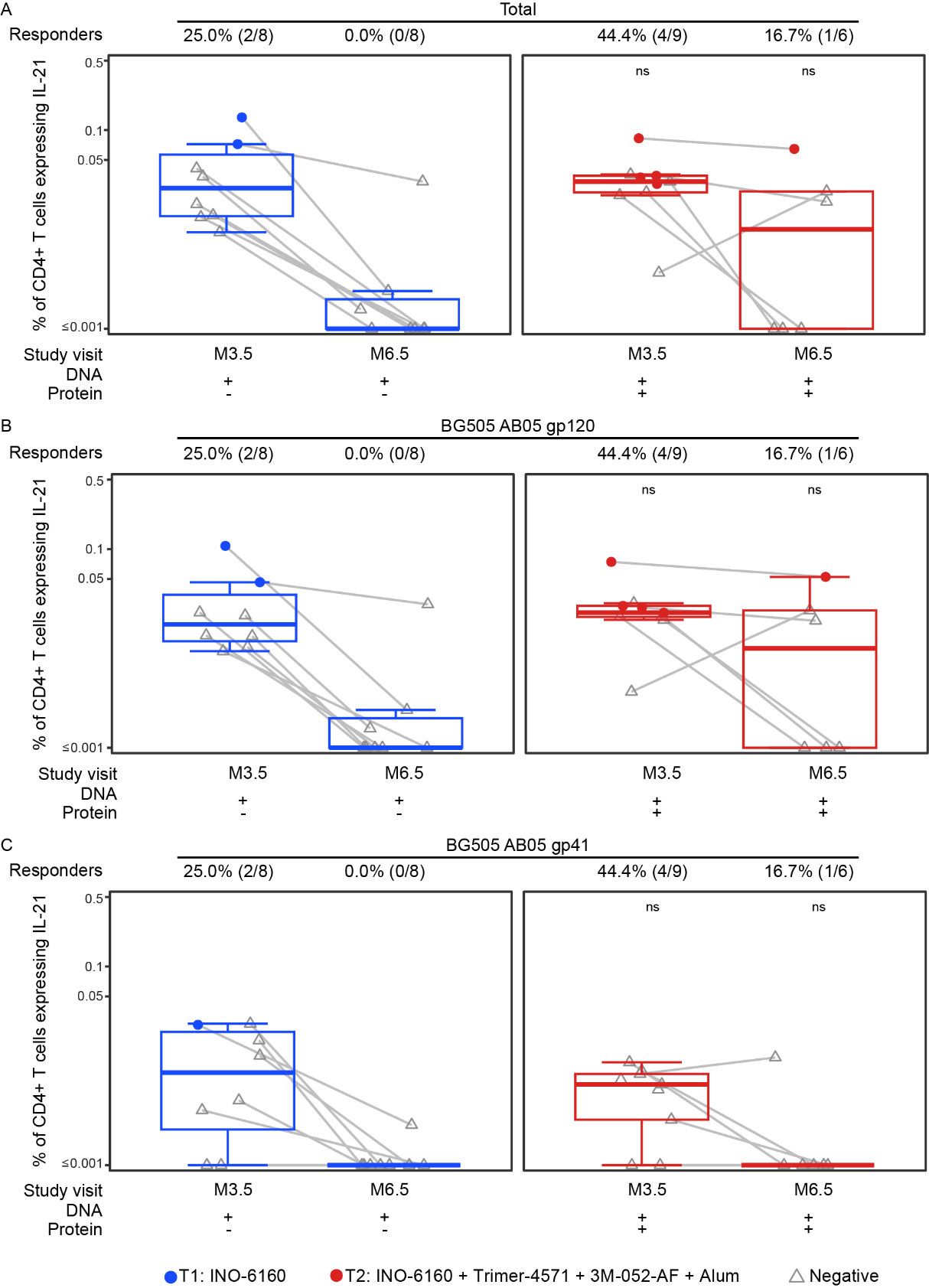


**Figure S7: Representative flow cytometry gating strategy for the 27-color ICS assay.** Previously cryopreserved PBMC from a healthy adult donor were stimulated with staphylococcal enterotoxin B (SEB) as the positive control (Part A) or with the peptide diluent (DMSO) as the negative control (Part B) for 6 hours, stained, and the data were subsequently acquired on a BD FACSymphony instrument. The upper two rows show the gating hierarchy to identify CD4+ and CD8+ T cells. Initial gating on time (seconds) to exclude any events early in collection due to pressure fluctuations, exclusion of aggregates (multiple plots, not shown), live cell gating, monocytes excluded based on the CD14-SS^lo^ gate, scatter gated on lymphocytes, singlets, B cells excluded as CD19-, CD3+/CD3- cells are gated against IFN-g, T cells are further defined as CD3+ CD16- on a CD3 vs CD16 plot (to exclude contaminating granulocytes that are CD16 and dim for CD3), gating out NK T cells on a CD56 vs CD16 plot, and finally, the T cells are defined by CD4 or CD8 expression on a CD4 vs CD8 plot. The next two rows show various functional and phenotyping markers for CD4+ T cells and the final two rows for CD8+ T cells. The functional marker gates are essentially based on one dimension (although visualized vs. another marker) and Boolean gates are created to identify cells expressing different combinations of markers. Most gates are copied, applied to all lineages, and cloned so that any changes to the gate on one lineage changes the gate on all lineages. For some populations, FMO controls were used to set the lower limits of the gates. Some gates are placed higher to improve the specificity, for example, for the functional markers based on the background as observed in the unstimulated controls.

| A  |
| --- |
| B  |

**Figure S8: Sex-stratified** **vaccine-specific B-cell responses against AB05 and Trimer 4571 at month 0 (M0), month 3.5 (M3.5) and month 6.5 (M6.5), baseline pre-vaccination, two weeks after the third and fourth vaccinations, respectively.** Responses were measured as the percent of IgG+ B cells staining for either Trimer AB05 or Trimer 4571 fluorescent probes. Responders are shown as % (N/total) for each study month. Female positive responses are shown in filled circles; male positive responders are shown as filled triangles. Female non-responders are shown in open circles; male non-responders are shown in open triangles. Box plots represent the distribution for all participants (the upper and lower quartiles and the median).

**
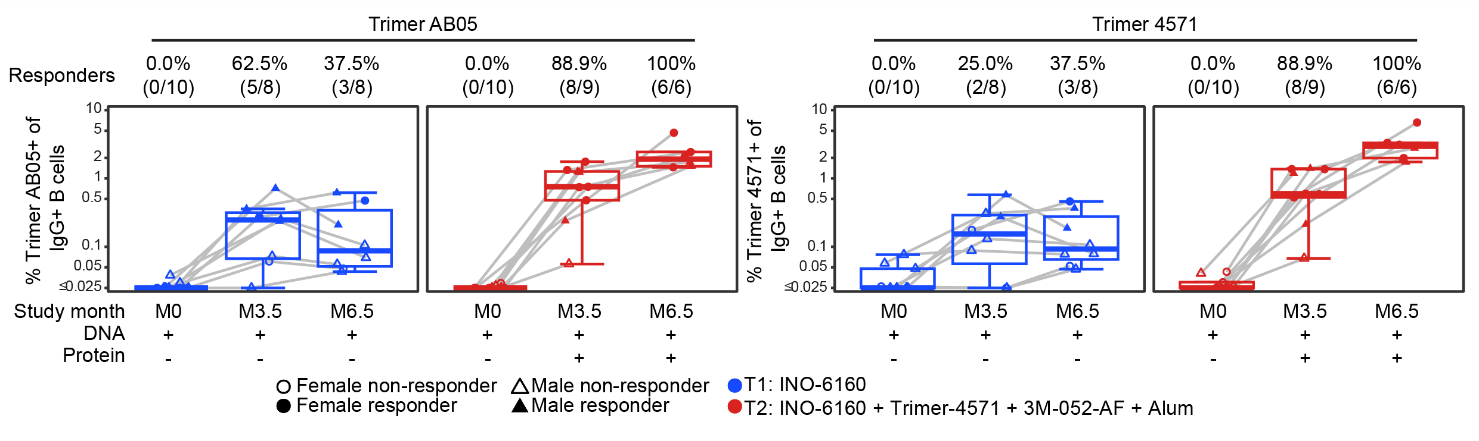
**

**Figure S9: B cell Representative Flow Cytometry Plot.** Previously cryopreserved PBMC from a healthy adult donor were stained, and the data were subsequently acquired on a BD FACSymphony instrument. Initial gating on time (seconds) to exclude any events early in collection due to pressure fluctuations, exclusion of aggregates (multiple plots, including FSC and SSC). Non-B cells and dead cells were excluded using markers CD3, CD14, and CD56 and a live/dead stain (“AVID”). B cells were identified by CD19 and CD20 expression; Memory B cells were defined as CD19+CD20+ lacking IgD. IgD- cells were further identified by isotype (either IgM+ or IgG+). HIV-1 Env–specific IgG+ memory B cells were then identified using fluorescent tetramers made by conjugating biotinylated Trimer AB05 gp140 and Trimer 4571 to SA-PE and SA-AF647.

**
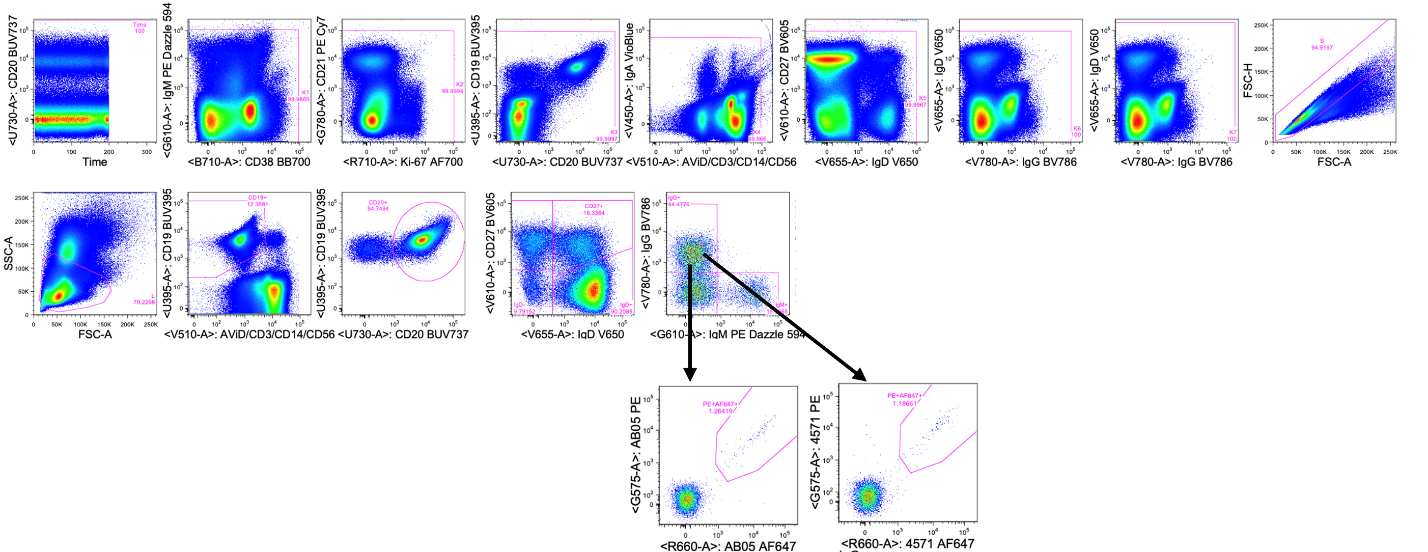
**

**Table S2: Reagents used in the 27-color flow cytometry ICS staining panel.**

| **Antibody** | **Manufacturer** | **Catalog Number** | **Clone** |
| --- | --- | --- | --- |
| Viability | Invitrogen | L34962 | N/A |
| TNF BUV395 | BD Biosciences | 563996 | MAb11 |
| CD45RA BUV496 | BD Biosciences | 624283/Custom | HI100 |
| CD19 BUV563 | BD Biosciences | 612916 | SJ25C1 |
| CD14 BUV661 | BD Biosciences | 624285/Custom | MΘP9 |
| CD154 BUV737 | BD Biosciences | 624286/Custom | TRAP1 |
| CD8 BUV805 | BD Biosciences | 612889 | SK1 |
| IFN-𝛾 V450 | BD Biosciences | 560371 | B27 |
| CD4 BV480 | BD Biosciences | 566104 | SK3 |
| CD16 BV570 | BioLegend | 302036 | 3G8 |
| CCR7 BV605 | BioLegend | 353224 | G043H7 |
| CD25 BV650 | BD Biosciences | 563719 | M-A251 |
| PD1 BV711 | BioLegend | 329927/329928^1^ | EH12.2H7 |
| CD56 BV750 | BioLegend | 362556 | 5.1H11 |
| CCR6 BV785 | BioLegend | 353422 | G034E3 |
| Perforin FITC | BioLegend | 353310 | B-D48 |
| IL-5 BB630* | BD Biosciences | 624294/Custom | TRFK5 |
| IL-13 BB630* | BD Biosciences | 624294/Custom | JES10-5A2 |
| Ki67 BB660 | BD Biosciences | 624295/Custom | B56 |
| IL-4 BB700 | BD Biosciences | 624381/Custom | MP4-25D2 |
| CXCR5 PE | BioLegend | 356904 | J252D4 |
| IL-21 PE-CF594 | BD Biosciences | 624352/Custom | 3A3-N2.1 |
| CXCR3 PE-Cy5 | BD Biosciences | 551128 | IC6/CXCR3 |
| FOXP3 PE-Cy5.5 | Invitrogen | 35-4776-42 | PCH101 |
| IL-17a PE-Cy7 | BioLegend | 512315 | BL168 |
| IL-2 APC | BioLegend | 500310 | MQ1-17H12 |
| Granzyme B Axl700 | BD Biosciences | 560213 | GB11 |
| CD3 APC-Fire750 | BioLegend | 300470 | UCHT1 |
| *IL-5 and IL-13 are detected on the same channel. | | | |
| ^1^Differences in catalog numbers are due to different volumes per vial. 329927 was used in the validation. | | | |
